# Chest CT features of coronavirus disease 2019 (COVID-19) in pediatric patients and its correlation with the clinical condition

**DOI:** 10.1186/s43055-022-00948-0

**Published:** 2023-01-03

**Authors:** Abeer El-Sayed Gabr, Abeer Maghawry Abdelhameed, Shaimaa Abdelsattar Mohammad, Eman Mahmoud Fouda, Shimaa Ahmed Maher, Samah Khalaf Fathallah, Mona Gamalludin Alsayed Muhammed Alkaphoury

**Affiliations:** 1 Department of Diagnostic and Interventional Radiology and Molecular Imaging, Faculty of Medicine, Ain Shams University, Cairo, Egypt; 2 Pediatric Department Faculty of Medicine, Ain Shams University, Cairo, Egypt

**Keywords:** COVID-19, CT severity score, ROC = receiver operating characteristic, LLL = left lower lobe, DCL = disturbed conscious level, DKA = diabetic ketoacidosis

## Abstract

**Background:**

All pediatric health organizations are concerned about the impact of coronavirus disease on children, especially on those with other comorbidities; fortunately, pediatric cases appear to be less severe than in adults (De Luca et al. in Pediatr Respir Rev 5:9–14, 2020). The purpose of this study is to characterize chest CT findings of children with and without comorbidities who had confirmed coronavirus disease (COVID-19) and to investigate the relation between chest CT findings and the clinical severity of COVID-19 pneumonia and their laboratory findings.

**Results:**

The study was conducted on 36 patients, 72.2% of whom had associated comorbidities. Twenty-three patients (63.88%) had abnormal CT findings. Consolidative patches were the most common radiological sign (55.6%) followed by ground glass opacities (50%). The lesions were bilateral (58.3%), having predominantly peripheral distribution (38.9%) with predominant left lower lobe affection (25%). Cases with clinically severe chest conditions had significantly more prevalent consolidative patches (*p* = 0.026) which show a higher CT density (*p* = 0.01) and a significantly higher CT severity score (SS) compared to other groups (*p* = 0.029). The cutoff of severity score 4/20 had 100% sensitivity and 78.12% specificity in the diagnosis of severe cases. There were no statistically significant differences between patients with or without comorbidities regarding CT-SS or any radiological signs.

**Conclusions:**

Consolidation was the most common radiological finding in children with COVID-19 and was more prevalent and denser in severe cases. The CT-SS may be used as a complementary tool for the evaluation of the severity of the chest condition. Chest CT-SS more than 4 can be used as an indicator of severe cases, yet no significant difference in CT-SS between patients with associated comorbidities or not.

## Background

Since the emergence of COVID-19 in December 2019, the number of children with confirmed COVID-19 infection has been increasing significantly [1]. In Egypt, more than a third of the population is made up of children [2]. Many studies had described the clinical and imaging features of COVID-19 in adults [3–6], but CT findings in children have been described in a small number of studies and a smaller number of patients [7, 8].

CT-SS is a semiquantitative measure used to evaluate the degree of lung involvement. It is a fast method that can help in the identification and evaluation of pediatric COVID patients and can be used as a complementary tool together with clinical and laboratory data to identify the severity of chest conditions [9, 10].

Severe disease is associated with higher CT severity scores in adults [11] but limited data about the relation between CT-SS and the clinical condition in the pediatric age group. As the imaging features of pediatric patients with COVID‐19 infection and its relation to the patient’s clinical condition were limited, we would like to present this study.

## Methods

### Study design and ethical approval

Ethical approval for this study was obtained from our institutional review board.

### Study population (eligibility criteria)

From June 1, 2020, to November 30, 2021, all children aged from 1 day to 18 years who have positive COVID-19 infection (confirmed by a nasopharyngeal swab and PCR) and available CT chest were initially included in the study. Children without available clinical data or having preexisting chronic lung condition that may hinder proper interpretation of pulmonary CT findings were excluded from the study.

All patients were hospitalized in our hospital either due to a chest condition or due to another cause.

#### Data collection

All patients’ data from medical records were reviewed, and the patients were categorized clinically according to their COVID-19 disease severity into mild, moderate, and severe cases [12]. Cases were considered mild when patients had mild upper respiratory symptoms (pharyngeal congestion, sore throat, and fever) or asymptomatic infection and had positive PCR tests and did not require hospitalization for their chest condition, moderate cases were those who were hospitalized in a simple ward (symptoms such as cough, fatigue, headache, and myalgia), while severe and critically ill patients were those who developed (hypoxia, respiratory failure or septic shock) necessitating ICU admission.

Demographic data (age and gender) of the patients were recorded together with an assessment of the presenting symptoms (respiratory, GIT, neurological, fever, and other symptoms), their duration, and identification whether there were associated comorbidities or not.

The duration between the positive PCR test results and CT acquisition was also recorded.

All important and available laboratory results were collected from the laboratory electronic database including CBC mainly lymphocytic count, CRP, LDH, and D-dimer in severe cases to exclude hypercoagulable state to start clexane and creatine kinase total and creatine kinase MB to exclude carditis.

### CT protocol and images acquisition

All patients were scanned by using a multidetector CT scanner (GE Optima CT660-128 slice). As the established protocol in our hospital, a non-contrast chest computed tomography is usually acquired (unless iodinated contrast is indicated in some clinical conditions (i.e., in oncological patients or to exclude pulmonary embolism in patients with elevated D-dimer)), checking serum creatinine and proper fasting if general anesthesia or contrast is required. The patient was scanned in the supine position and centered in the CT gantry. Positioning the arms above the head is preferable. Scanning time was 2.5–20 s, and scans were obtained from the lung apex to the posterior recess of the lung to cover the entire lung parenchyma.

In two of the studied cases, contrast media was needed, one of them was suspected to have malignancy and the other had hemophagocytic lymphohistiocytosis (HLH) activity for better identification of mediastinal lymph nodes. A dual injector was used for contrast injection: Syringe A: nonionic contrast (1.5 ml/kg) and Syringe B: 20 ml saline. A nonionic contrast agent was injected through a peripheral venous line by using a flow rate (1.5–2.5 ml/sec) followed by 20 ml saline.

All imaging data were reconstructed by use of a medium sharp reconstruction algorithm with a slice thickness of 0.625–5 mm and then were sent to the picture archiving and communication system (PACS) for image analysis.

### CT image evaluation

Image analysis was performed by using (PACS). Two experienced radiologists (with 5 and 12 years of experience) reviewed the CT images independently; they were blinded to the clinical data. The disagreement was settled in consensus with the help of a third experienced radiologist with 20 years of experience.

CT images were evaluated using a lung window with a window level of − 600 HU and window width of 1500 HU, and the soft tissue window with a window level of 40 HU and window width of 300 HU, using both axial CT images and multiplanar reconstruction images.

Different CT findings were described according to the Fleischner society glossary of terms [13]. The prevalence was documented (ground‐glass opacities (GGO), consolidation, halo sign, reverse halo sign, vascular thickening, vascular sign, solid nodules, traction bronchiectasis, peribronchial thickening, perilobular thickening, subpleural arcade opacities, fibrous strips, spider web opacities, and crazy paving). The distribution of different lesions according to their location (central, peripheral, patchy, diffuse), laterality (unilateral or bilateral), the number of lobes affected (single or multiple lobes), and the predominantly affected lobe were assessed. The presence of pleural effusion and lymph nodes was also documented [14].

Multiple scores were used to calculate the degree of lung involvement; in this study, we focused on the CT severity score introduced by Chung et al. [3], the score ranging from 0 to 4 was determined for each lobe with 0 indicating no involvement, 1 indicating less than 25% involvement, 2 indicating 25–50% involvement, 3 indicating 51–75% involvement, 4 indicating 76–100% involvement, so total score ranged from 0 to 20 [5].

According to the CT predominant feature, the disease stage was determined: stage 0: normal study, GGO represents stage 1, crazy paving and consolidation represent stage 2, consolidation represents stage 3 and fibrotic bands represent stage 4 [15].

The density of the densest lesion in the CT was calculated to assess whether there is a correlation between the density of consolidation and the severity of the disease.

The Radiology Society of North America (RSNA) category and coronavirus disease 2019 (COVID-19) Reporting and Data System (CORADS) score were documented for all cases.

### Statistical analysis

Different groups of clinical severity (mild, moderate, and severe) were compared regarding the prevalence of different radiological signs, their CT-SS, RSNA categories, CORADS, disease stage, and density of consolidation.

And the CT-SS was correlated with RSNA categories and also with all performed laboratories.

ROC curve was used to test the ability of CT-SS to differentiate between the mild and moderate groups and the severe group.

Patients with and without associated comorbidities were compared regarding the prevalence of different radiological signs (consolidation, GG opacities, halo sign, and vascular sign) as well as severity scores.

## Results

### Clinical analysis

The included 36 patients had a median (IQR) age of 1.38–9.5 years and 50% were males. Twenty-six patients (72.2%) had associated comorbidities. Eleven patients (25%) had malignancies (hematological including leukemia, lymphoma, and HLH and non-hematological as Medulloblastoma and Ewing sarcoma), and 4 patients (11.1%) had congenital diseases (heart diseases, laryngomalacia, and multiple congenital anomalies), genetic diseases as an inborn error of metabolism. Other comorbidities include diabetes mellitus, chronic kidney disease, cerebral palsy, epilepsy, and Glanzmann disease.

Fever was the most common presenting symptom (63%) followed by dyspnea (38.9%), cough (27.8%), vomiting (19.4%), and disturbed conscious level (16.7%) as given in Table 1.

**Table 1:** Presenting clinical symptoms and signs

	No	%
*Respiratory symptoms*		
Cough	10	27.8
Dyspnea	14	38.9
Wheezes	2	5.6
Chest pain	1	2.8
*Neurological symptoms*		
Convulsions	2	5.6
Abnormal movement	2	5.6
Squint	3	8.3
DCL	6	16.7
Headache	1	2.8
Motor weakness	1	2.8
*GIT symptoms*		
Vomiting	7	19.4
Diarrhea	4	11.1
Distension	3	8.3
Anorexia	3	8.3
WIGHT LOSS	3	8.3
Nausea	2	5.6
PALLOR	2	5.6
Others (abdominal pain, constipation)	2	5.6

While (63.9%) of the patients were mild, the moderate group represented 25%, and the severe group represented 11.1%.

There was no significant correlation between the patient’s demographic data including age, sex, and the severity of the clinical picture.

Also, there was no significant correlation between clinical severity classification (mild, moderate, and severe) as regards the duration of fever, vomiting duration between the onset of the complaint and date of imaging, and presence of comorbidities or not.

### Radiological analysis

All moderate and severe cases underwent chest CT due to their chest condition, and the selected mild cases underwent CT due to causes related to their underlying comorbidities.

**Fig. 1:**
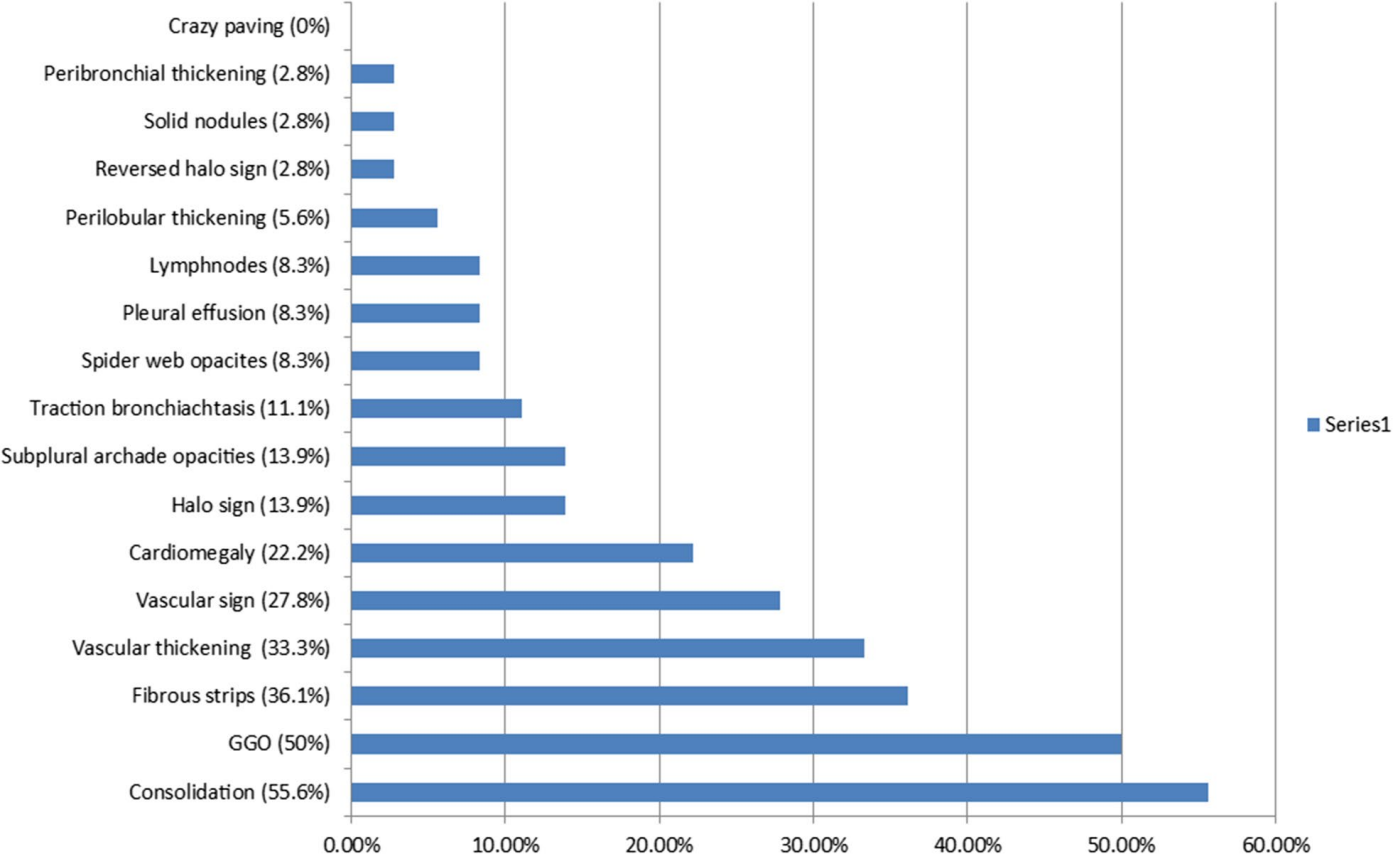
Representing the percentage of CT radiological signs

**Fig. 2:**
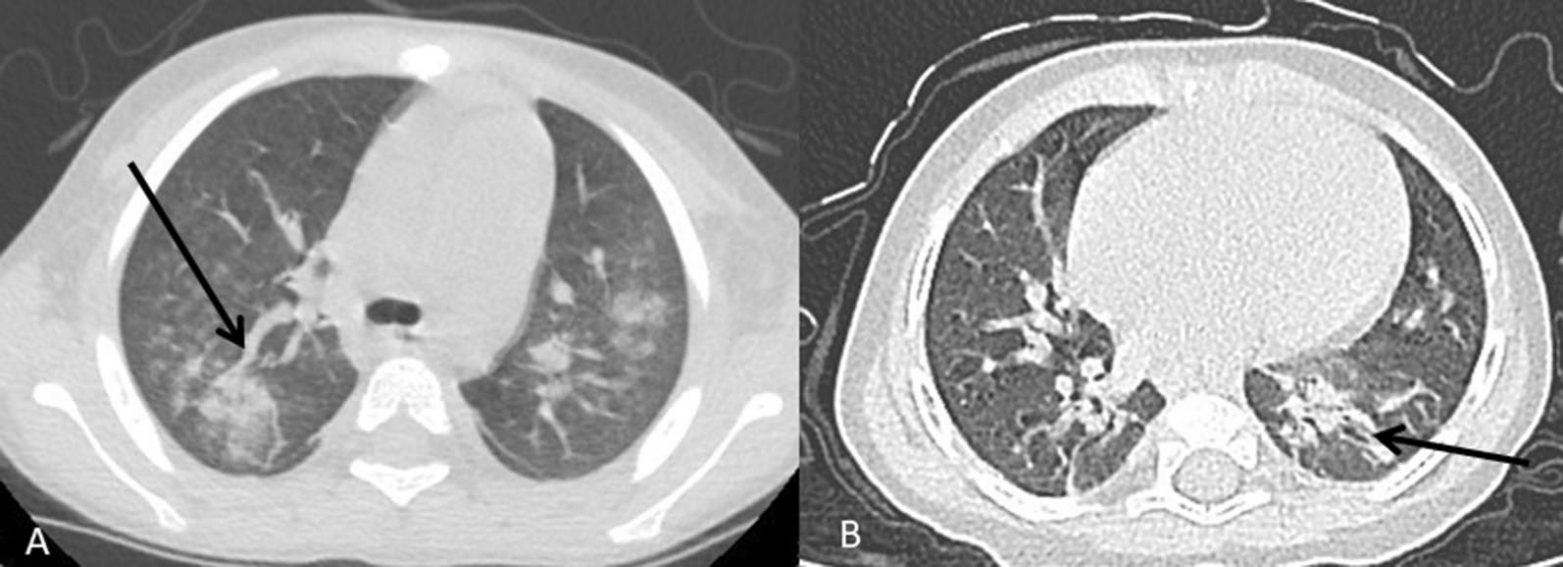
**A** A 7-year-old male patient with vascular sign (arrow) and **B** an 8-month-old male patient with left lung traction bronchiectasis (arrow)

Twelve patients (33.3%) had normal CT chest findings. Consolidation patches were the most common radiological sign. It was found in 20 patients (55.6%), followed by GGO which was found in 18 patients (50%). Pleural effusion and mediastinal lymph nodes were seen in 8.3% of the cases, cardiomegaly was noted in 22.2% of the cases, and other signs are presented in Figs. 1 and 2.

The lesions were bilateral (58.3%), multilobar in 61.1%, predominant lobe affected was the LLL (25%) followed by RLL (19.4%) with prominent peripheral distribution (38.9%).

Consolidative patches were more prevalent in the severe group (100%) compared to the mild group (39%) with a statistically significant correlation (*p* = 0.026) yet no significant correlation between these groups as regards ground glass opacities, halo sign, and vascular sign.

The median density of consolidation was higher in the severe group (54.5HU) compared to the moderate (37.2HU) and mild (− 201HU) groups with a statistically significant correlation (*p* = 0.01).

**Fig. 3:**
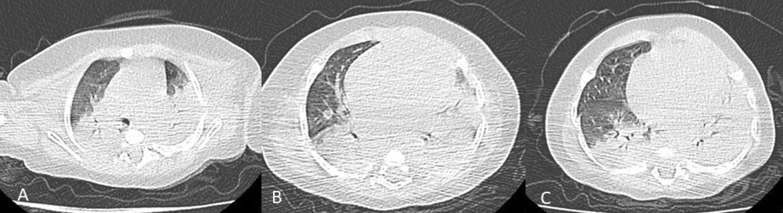
A 6-year-old male patient with **A** RUL (25–50%) score 2, LUL (50–75%) score 3, **B** RML (0–25%) score 1, **C** RLL (50–75%) score 3 and LLL (75–100%) score 4 with total score 13/20

**Fig. 4:**
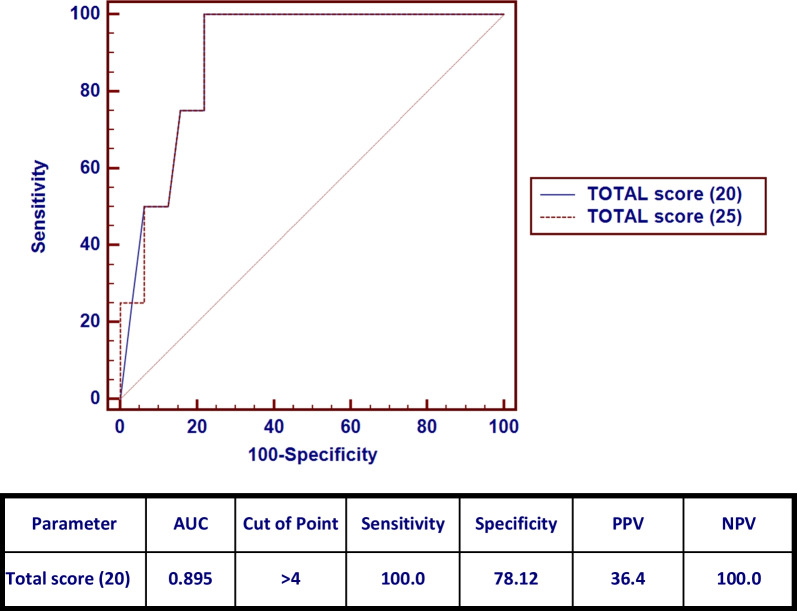
ROC curve of CT-SS with a total score of 20 as a predictor of severity of chest condition

CT severity score calculated with a total score of 20 had a median score of 3.5 and IQR (0–8) (Fig. 3). Cases with clinically severe chest conditions had a statistically significant higher CT score compared to other groups (*p* = 0.029). Of the total CT-SS score (20), the cutoff of 4 had 100% sensitivity and 78.12% specificity in the diagnosis of severe cases (Fig. 4).

**Fig. 5:**
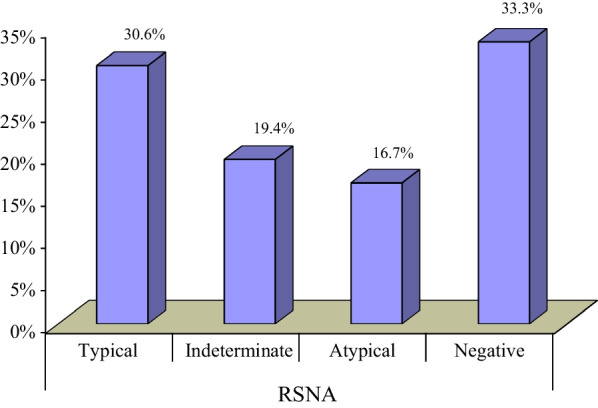
Percentage of cases in each RSNA category

**Fig. 6:**
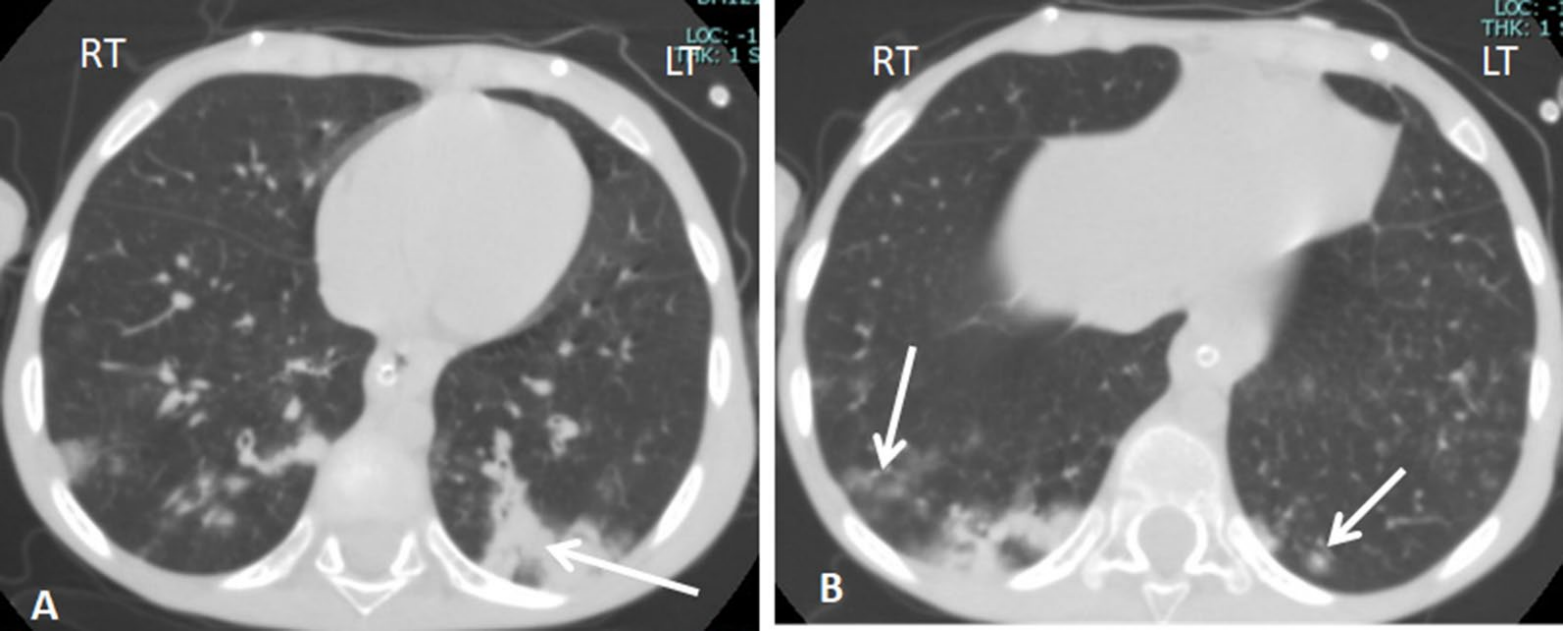
A 5-year-old female child patient, a known case of medulloblastoma presented with fever and dyspnea for 7-day duration; she had severe respiratory distress and was PCR positive. CT axial images showing bilateral scattered peripheral subpleural consolidations (arrow in **A**), (arrows in **B**) ground glass opacities in the right lung and halo sign in the left; picture is typical CT findings for COVID-19, CT severity score 6/20

**Fig. 7:**
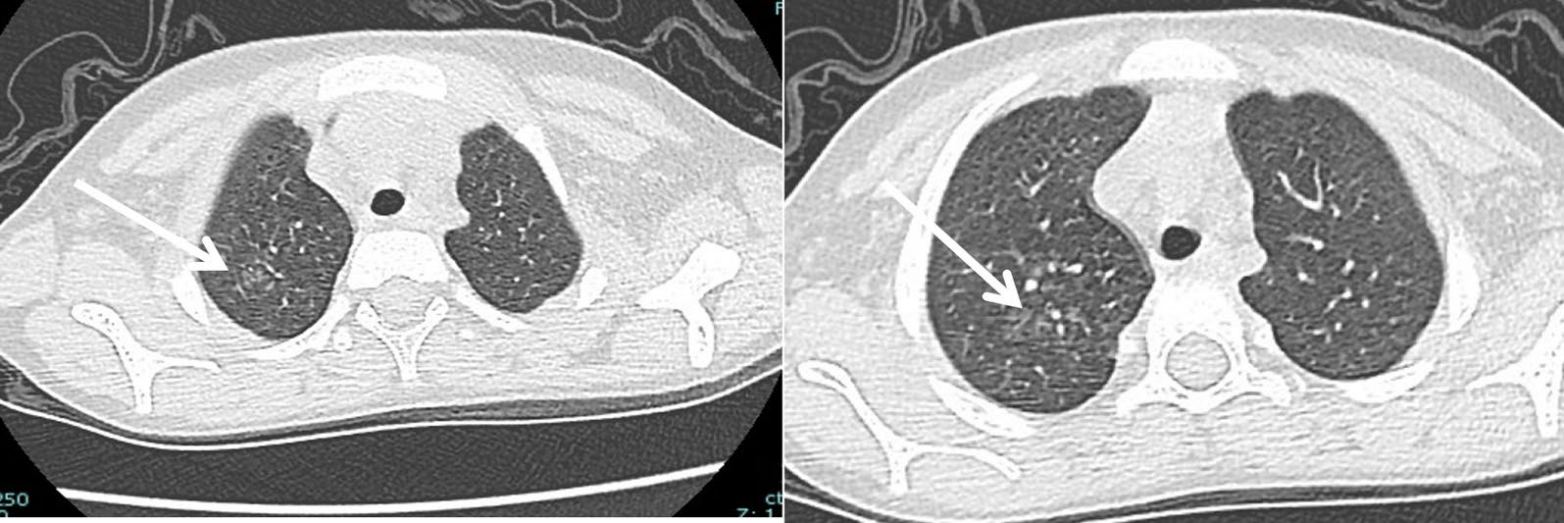
A 12-year-old male child, known case of leukemia, presented with fever for 4-day duration with mild respiratory manifestation and PCR positive; CT shows unilateral upper lobar centrilobular ground glassing opacities, CT picture is indeterminate for COVID-19, and CT severity score was 3/20

**Fig. 8:**
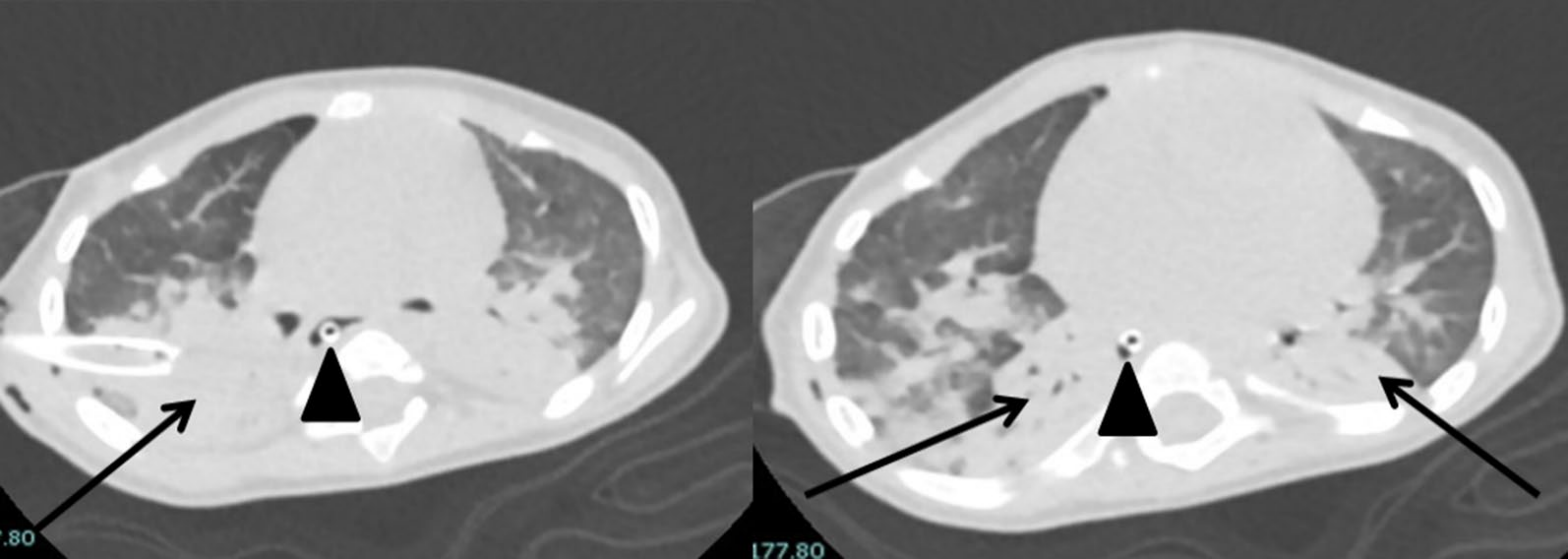
A 8-month-old female baby, known case of Down syndrome and congenital heart disease (VSD, ASD, and PDA), presented by acute dyspnea with severe respiratory manifestation. CT shows bilateral upper and lower lobar posterior consolidation and atelectasis (note chest tube is seen in the right lung due to pneumothorax (resolved)), picture atypical for COVID, and CT severity score was 15/20. *Note*: the nasogastric tube is seen slightly deviated to the right (black triangle)

According to RSNA classification, 12 cases (33.3%) were negative, 11 cases (30.6%) were typical, and 6 cases (16.6%) were atypical which was found to have the highest CT-SS (*p* = 0.000) (Figs. 5, 6, 7, 8).

**Fig. 9:**
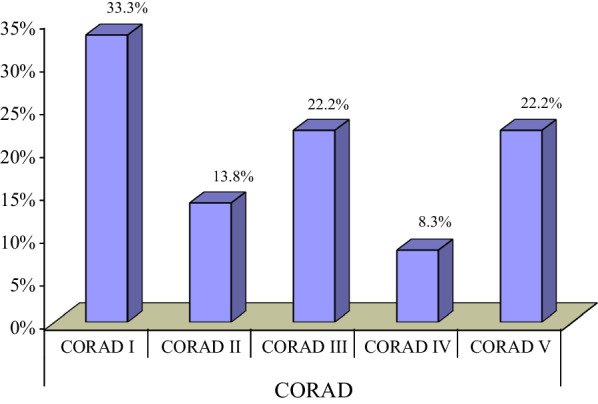
Percentage of cases in each CORAD

And according to CORADS classification, most cases were CORAD I (33.3%) followed by CORAD V and III yet no correlation between it and the CT-SS or classes of clinical severity (Fig. 9).

According to CT COVID-19 staging introduced by Pan et al. [15],13 patients (36.1%) were in stage 3 followed by 12 patients (33.3%) in disease stage 0 while late stages (stage 4) were only 6 patients (16.7%), also no correlation between CT staging and the CT-SS nor classes of clinical severity.

No statistically significant correlation between the presence of comorbidities and CT-SS nor any radiological signs (consolidation, GG opacities, halo sign, vascular sign).

There was no significant correlation between the CT-SS and the duration between the onset of the disease and the timing of CT.

#### Laboratory result analysis

As shown in Table 2, leukopenia was noted in 13 patients (38.2%) of cases while leukocytosis was noted in 8 patients (23.5%), and CT-SS was found to be higher in cases with leukocytosis compared to those having leucopenia with median CT-SS 3.5 and 0, respectively (*p* = 0.044). Also, direct bilirubin was high in 7 patients (28%) and there were associated significantly higher CT-SS in those patients compared to patients with normal direct bilirubin with median CT-SS of 13 and 2.5, respectively (*p* = 0.027).

**Table 2:** Presenting important lab results done to the patients

	No. = 36
Leukopenia	13 (38.2%)
Leukocytosis	8 (23.5%)
Lymphopenia	18 (52.9%)
Lymphocytosis	2 (5.9%)
Low hemoglobin level	26 (76.5%)
Thrombocytopenia	11 (32.4%)
Thrombocytosis	5 (14.7%)
High ALT level	10 (31.3%)
High AST level	11 (36.7%)
High total bilirubin level	3 (12.0%)
High direct bilirubin level	7 (28.0%)
High CRP Level	20 (71.4%)
Low ferritin level	7 (28.0%)
High ferritin level	18 (72.0%)
High D-dimer level	16 (72.7%)
High troponin level	4 (30.8%)
High CK total level	5 (23.8%)
High CK-MB level	9 (47.4%)
High LDH level	15 (51.7%)

There was no statistically significant correlation between CT severity score and lymphocytic, platelet count, hemoglobin level, ALT, AST, total bilirubin, CRP, ferritin, D-dimer, troponin, CK total, CK-MB, and LDH level.

## Discussion

Coronavirus disease was considered a major health problem. In May 2022, about 13.3 million reported pediatric COVID-19 cases which represent 19.0% of all cases in the USA [16]. Laboratory testing was the standard for the diagnosis of COVID-19 pneumonia, but the supply of laboratory kits cannot meet the demand of the increasing number of suspected cases, especially in developing countries as well as the presence of false negative results [4]. This increases the demand for better characterization of radiological findings in CT and a semiquantitative assessment of the severity score and its correlation with the clinical condition.

COVID-19 radiological chest manifestations and their relation to the clinical condition have been discussed numerously in adults, but limited data are available in the pediatric population.

**Fig. 10:**
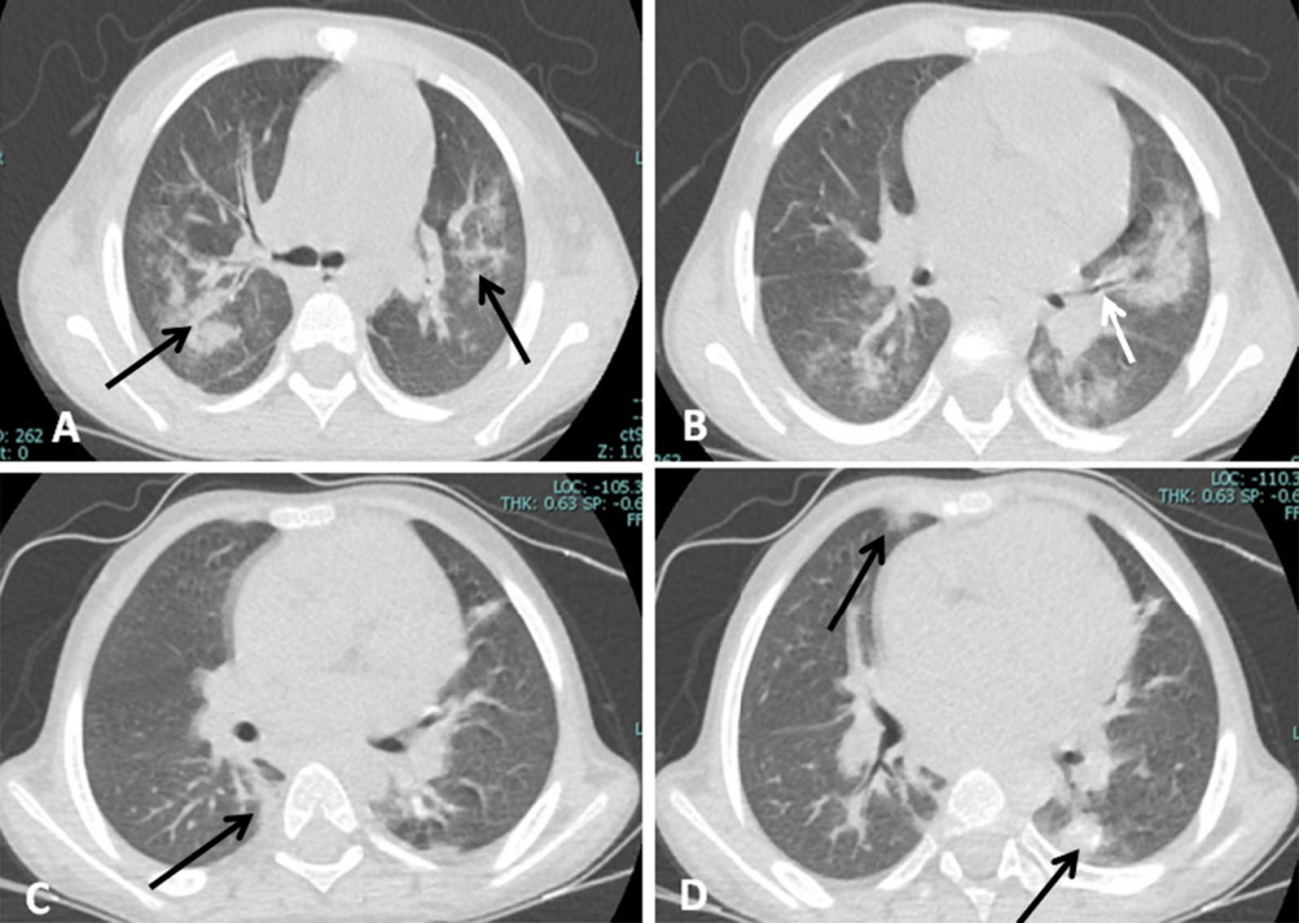
(**A**, **B**) A 7-year-old male patient known case of Glanzman disease presented with bleeding from the nose with mild respiratory manifestation and PCR positive for COVID-19. CT axial images, showing bilateral central consolidation with areas of centrilobular nodular infiltrates of air spaces (black arrows); findings are indeterminate for COVID-19, and CT severity score was 10/20. An air bronchogram is seen in the left lung (white arrow). (**C**, **D**) A 4-year-old male patient diagnosed with HLH presented with a fever. CT axial images, showing bilateral peripheral ground glass opacities and consolidations (black arrows); findings are typical for COVID-19, and CT severity score was 4/20. Cardiomegaly is also noted

More than 70% of our patients had associated comorbidities, none of them were affecting the chest CT finding except one case diagnosed with Glanzmann disease and presented with epistaxis, and CT chest shows bilateral consolidation more central than peripheral which is an indeterminate probability for COVID-19 which could be explained by pulmonary hemorrhage (Fig. 10).

In our study, consolidative patches were the most prominent feature followed by GGO, typically of peripheral distribution, and bilateral multilobar with lower lobe predominance, which is different from most other studies [17, 18] that show GGO as the predominant CT feature. We primarily explain this by the presence of a co-bacterial infection due to reduced immunity in most of our patients with other comorbidities, but there was no statistically significant difference in consolidation between patients with comorbidities or not. This could be explained that consolidative pattern was significantly higher in moderate and severe groups which represent one-third of our cases, this is consistent with a study done on 31 severe COVID-19 pediatric patients with consolidative patches were the most predominant CT feature [19].

None of the severe cases were CT negative, and only one case in the moderate group was CT negative; this could be explained by the short duration between the onset of symptoms and CT acquisition while 52% of mild cases had abnormal CT chest which represents the high sensitivity of the CT.

CT severity score is a semiquantitative measure used to assess the degree of lung involvement; it was significantly higher in severe patients. A severity score cutoff value of 4 (25%) lung involvement is used by CT-SS to identify severe cases; however, Romeih et al. [20] stated that the cutoff point for severe cases was 6.5 (32%) but our CT-SS cutoff point has a higher sensitivity and specificity 100% compared to 90.9% and 78.12% compared to 69%, respectively.

According to our results, we should consider patients with a total CT severity above 4 as a predictor of the severe course of the disease.

Of all our patients, fever was the most common patient’s symptom followed by respiratory symptoms, which agreed with other studies in pediatrics and adults [8, 17].

Few cases were presented with DCL, most of them had other comorbidities (diabetes mellitus and presented by DKA, multiple congenital anomalies and intracranial hemorrhage in CT, epilepsy, quadriplegic CP, and leukemia), one patient showed no associated comorbidities, so we cannot determine whether DCL was due to COVID-associated vacuities or not.

In our study, leukopenia was noted in 38.2% of cases while leukocytosis was in 23.5%; the CT-SS was found to be higher in cases with leukocytosis compared to those having leucopenia which is inconsistent with a pooled analysis and review [21] which stated that there is no specific pattern of leucocyte abnormalities in pediatric COVID-19 population in relation to the clinical severity; this could be explained by the high percentage of severe cases in our study compared to other papers, and those sever cases are more liable to secondary bacterial infection in the chest or any other site in the body which leads to increased leukocytosis.

Forty-seven percent of our cases had elevated CK-MB consistent with the pooled analysis and review on laboratory abnormalities of pediatric COVID-19 [3], and one-third of these cases had congenital heart diseases and other comorbidities including multiple congenital anomalies, HLH activity, leukemia, DKA, and epilepsy. Only one case had no associated comorbidities and may represent cardiac injury; also 70% of patients with elevated CK-MB had severe general conditions, so this biomarker could represent an inflammatory biomarker.

A significant correlation between the CT-SS and elevated direct bilirubin was found in our cases, which is consistent with other papers, stating that the level of direct bilirubin is one of the best markers for diagnosing the severity of the disease in children [22].

## Conclusions

Consolidation was the most common radiological finding in children with COVID-19, and they were more prevalent and denser in severe cases.

The CT-SS may be used as a complementary tool for the evaluation of the severity of the chest condition. Chest CT-SS more than 4 can be used as an indicator of severe cases.

There was no significant difference in CT-SS between patients with associated comorbidities or not.

## Data Availability

The datasets used and analyzed during the current study are available from the corresponding author on reasonable request. Competing interests: No financial or non-financial competing interests.

## Abbreviations

COVID-19: Coronavirus disease 2019
CT-SS: CT severity score
ROC: Receiver operating characteristic
LLL: Left lower lobe
DCL: Disturbed conscious level
DKA: Diabetic ketoacidosis
CORADS: Coronavirus disease 2019 (COVID-19) reporting and data system
RSNA: Radiology society of North America
HU: Hounsfield unit
HLH: Hemophagocytic lymphohistiocytosis
